# Signal Identification of Gear Vibration in Engine-Gearbox Systems Based on Auto-Regression and Optimized Resonance-Based Signal Sparse Decomposition

**DOI:** 10.3390/s21051868

**Published:** 2021-03-07

**Authors:** Yuanyuan Huang, Shuiguang Tong, Zheming Tong, Feiyun Cong

**Affiliations:** 1 The State Key Laboratory of Fluid Power and Mechatronic Systems, Zhejiang University, No. 38, Zheda Road, Hangzhou 310027, China; huang_yy@zju.edu.cn (Y.H.); cetongsg@zju.edu.cn (S.T.); fycong@zju.edu.cn (F.C.); 2 School of Mechanical Engineering, Zhejiang University, No. 38, Zheda Road, Hangzhou 310027, China

**Keywords:** gear vibration, signal identification, auto-regression (AR), resonance-based signal sparse decomposition (RSSD), engine-gearbox

## Abstract

As an essential part of the transmission system, gearboxes are considered as a major source of vibration. Signal identification of gear vibration is necessary for online monitoring of the mechanical systems. However, in engine-gearbox systems, the ignition impact of the engine is strong, so that the gear vibration is generally submerged. To overcome this issue, the resonance-based signal sparse decomposition (RSSD) method is used in this paper based on different oscillatory behaviors of the gear meshing impact and the engine ignition impact. To improve the accuracy of RSSD under interferences, the meshing frequency energy ratio (MF–ER) index is introduced into RSSD to adaptively choose the decomposition parameters. Before applying the RSSD method, the auto-regression (AR) model is used as a pre-whitening step to eliminate the normal gear meshing vibration, which improves the decomposition performance of RSSD. The effectiveness of the proposed AR-ORSSD (AR-based optimized RSSD) algorithm is tested using both simulated signals and measured vibration signals from an engine-gearbox system in a forklift. Comparisons were made with the RSSD algorithm based on a genetic algorithm. Experimental results indicate that the AR-ORSSD algorithm is superior at identifying gear vibration signals especially when under strong interferences.

## 1. Introduction

Gearboxes are one of the most fundamental and crucial components in a wide range of mechanical systems, such as automobiles, ships, aircrafts, turbines, and so on [1,2]. The gear vibration is regarded as one of the main factors affecting the operating performance of the system. As a key element in rotating machines, it is important to extract the gearbox vibration part from the mechanical system signal to assess the health state of the gearbox. In general, meshing frequency is considered as the most representative characteristic signal of gear vibration. Therefore, the identification of gear meshing frequency is necessary for the online condition monitoring of the mechanical system [3]. However, in the engine-gearbox system, the ignition impact of the engine is so strong that the gear meshing frequency is generally hard to identify. Besides, in some special cases, the meshing frequency part may be modulated to the higher frequency band as the meshing impacts. Therefore, measurements should be taken to extract gear meshing frequency.

There is a great number of algorithms invented to extract gear characteristic signals, such as envelope demodulation [4,5,6], spectrum kurtosis [7,8,9], empirical mode decomposition (EMD) [10,11,12,13], wavelet transform [14,15,16], intelligent deep learning [17,18,19], and so on. The intelligent deep learning method has attracted much attention nowadays, however, it has some drawbacks that hinder its development. Firstly, it needs massive samples; then, the deep learning model usually does not have a specific physical meaning; finally, the training process is time-consuming [20]. Other traditional methods are used to process signals based on frequency. When a local fault occurs in the gear, the fault characteristic signal presents different center frequencies and bandwidths in the frequency domain. Therefore, the fault characteristic signal can be easily identified by these methods. However, for a healthy gearbox in a complicated system, the frequency components are coupled. The vibration signals of different components may have similar frequency characteristics. Moreover, the frequency domain may be complex due to various interferences. Thus, these methods are not suitable for the signal identification of a healthy gearbox in a complicated system.

The tunable-Q wavelet transform (TQWT) method was first proposed by Selenick in 2011 [21]. Based on this, the resonance-based signal sparse decomposition (RSSD) algorithm was presented [22]. Using the RSSD method, the signals can be decomposed into two different components with high and low *Q*-factors respectively based on oscillatory behavior rather than on frequency. Many researchers have since introduced RSSD into the field of rotating machinery fault diagnosis [23,24]. Wang et al. [25] extracted the early fault feature of a rolling bearing by combing RSSD with ensemble empirical mode decomposition (EEMD). Cai et al. [26] used RSSD to diagnose localized faults in gearboxes based on their oscillatory behaviors. Yan et al. [27] proposed a time-frequency signature using RSSD, manifold learning, and phase space reconstruction for ship-radiated noise identification. These researches all employed the original RSSD technique, where the determination of the decomposition parameters is quite arbitrary, relying mostly on prior information. According to some references [28,29], the selection of the *Q*-factors plays a crucial part in the performance of RSSD. Therefore, quite a few researchers have paid attention to optimizing the decomposition parameters. Huang et al. [30] pioneered the application of a genetic algorithm (GA) for parameter optimization and attracted a lot of attention. Zhang et al. [31] combined RSSD with some other techniques to achieve compound fault diagnoses in gearboxes. In their research, GA was employed to obtain the optimal parameters for RSSD. Zhang et al. [32] presented a novel method called improved singular value decomposition (ISVD) with RSSD to detect train bearing faults with wayside acoustic signals. The GA was also applied to maximize the kurtosis of the low *Q*-factor part. Chen et al. [33] put up an early fault diagnosis algorithm based on wavelet transformation and RSSD by optimizing the quality factor using GA and sub-band reconstruction. Zhang et al. [34] improved the RSSD method based on GA and demodulation analysis. Apart from GA, some other algorithms have also been proposed for the parameter optimization of RSSD. Chai et al. [35] optimized the decomposition parameters of RSSD using an artificial bee colony algorithm. Zhang et al. [36] and Wang et al. [37] both used the stepwise optimization strategy to obtain better RSSD results.

The auto-regression (AR) model is a commonly used time-series technique. It is appropriate for modeling deterministic components with sharp peaks in the frequency spectrum [38]. Rantala and Suoranta [39] first applied the AR model to monitor gear state using residual signals. Later, advances in the AR model for fault diagnosis and prognosis were made by researchers. For fault diagnosis, Cheng et al. [40] combined the AR model with empirical mode decomposition to extract the fault feature of roller bearings. Randall et al. [41] enhanced the ability of the AR model with minimum entropy deconvolution for gear fault diagnosis. Li et al. [42] developed a new technique for multi-fault diagnoses in gears based on a combined AR model, wavelet transformation, and principal component analysis. For gearbox prognosis, Zhan et al. [43] established a statistical indicator based on AR model residuals to monitor the gear state. Cong et al. [44] combined the AR model with spectral kurtosis for the early fault diagnosis and prognosis of bearings. Huang et al. [45] used the phase space warping method enhanced by the AR model to track bearing faults. In these researches, the AR model is used as a pre-processing step to obtain the residual signals. This function will also be employed in our research.

The references above all aim to extract the fault features of gearboxes when local faults occur. Few papers have tried to identify the vibration signal of a healthy gearbox. However, researches have indicated that for a complex mechanical system, the gear vibration signal is sometimes difficult to identify due to the interference of other components [46,47]. Taking the engine-gearbox system as an example, this paper presents a novel algorithm called AR-ORSSD (AR model-based optimized RSSD) for gear vibration signal identification. In the engine-gearbox system, there are two kinds of impacts: the gear meshing impact and the engine ignition impact. They can be successfully separated using the RSSD method due to different oscillatory behaviors. However, the accuracy of RSSD is limited under strong interferences. To improve the decomposition performance of RSSD, this paper introduces the MF–ER index to adaptively select the optimal *Q*-factors, which play an important role in RSSD accuracy. Considering the existence of gear meshing vibration, the AR model is incorporated into RSSD as a pre-whitening process. Both simulation and field experiments were carried out to assess the performance of the AR-ORSSD algorithm. Comparisons were made with the popular RSSD algorithm optimized by genetic algorithm (GA). Experimental results indicate that the AR-ORSSD algorithm is superior at identifying gear vibration signals especially when under strong interference.

The paper is arranged as follows: Section 2 describes the whole algorithm of AR-ORSSD. The simulation and field experiments are provided in Section 3 and Section 4, respectively. Finally, the main content of the paper is concluded in Section 5.

## 2. Methodology: Auto-Regression Model-Based Optimized Resonance-Based Signal Sparse Decomposition (AR-ORSSD)

**Figure 1:**
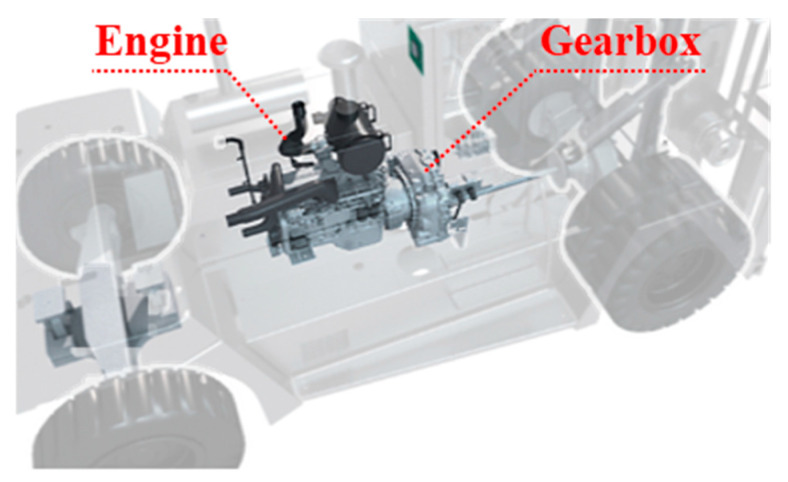
The engine-gearbox transmission system of a forklift.

According to the engine-gearbox transmission system shown in Figure 1, the power produced by the engine is transmitted to the gearbox. Therefore, the vibration signal obtained from the gearbox is interfered by the engine ignition impact signal transmitted through the transmission path. Since the ignition impact of the engine is strong, the gear vibration signal is always covered and hard to identify. In this section, the auto-regression model-based optimized resonance-based signal sparse decomposition (AR-ORSSD) method is proposed to extract gear meshing frequency.

### 2.1. Pre-Whitening with the AR Model

**Figure 2:**
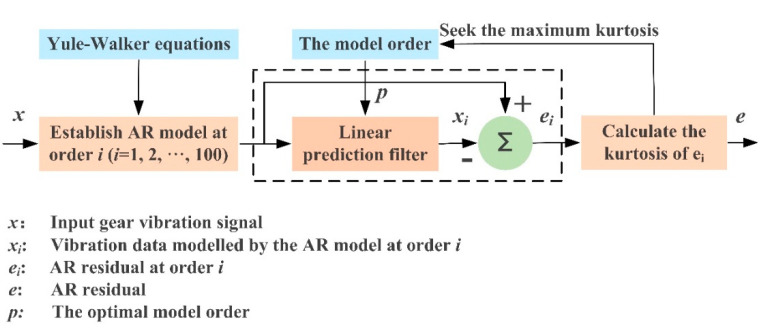
The flow chart of using the auto-regression (AR) model for pre-whitening.

During the gear meshing process, meshing impacts will be produced between meshing tooth pairs [3]. Therefore, the gear vibration signal includes normal gear meshing vibration and gear meshing impacts. The AR model is a statistical way to deal with time series, which is appropriate for modeling gear meshing vibration [38]. The AR model can be approximated by Equation (1), where the value at time t is the linear combination of the values at previous times plus an error term,

(1) $$x_{t}\;=\;\overset{n}{\underset{i\;=\;1}{\sum }}a_{i}x_{t-i}+\varepsilon_{t}$$

where $x_{t}$, and $x_{t-i}$ are the data points at time t, t−i respectively, n is the model order, $a_{i}$ denotes ith coefficient of the AR model and $\varepsilon_{t}$ is residual error. In our algorithm, the AR model coefficients are estimated using the Yule–Walker equations (YWEs) [38], the model order is selected by seeking the maximum kurtosis of $\varepsilon_{t}$ [48]. Therefore, the AR residual error only contains the remaining gear meshing impacts. The flow chart of using the AR model for pre-whitening is plotted in Figure 2.

### 2.2. Optimized Resonance-Based Signal Sparse Decomposition Based on Meshing Frequency Amplitude Ratio

**Figure 3:**
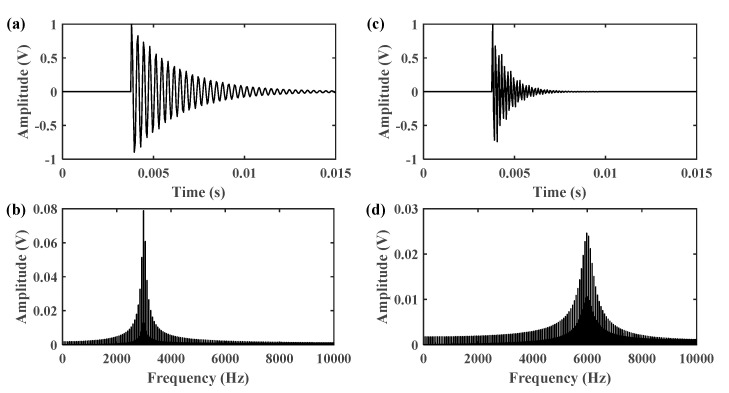
The time domain impulses and frequency spectrums of: (**a**,**b**) the gear meshing impact; (**c**,**d**) the engine ignition impact.

In the engine-gearbox system, there are two sources of impulses: the engine ignition impact and the gear meshing impact. However, the two impacts present different oscillatory behaviors and they can be found in the high resonance component and low resonance component, respectively, by using the RSSD method. The reason is that the gear meshing impact has better frequency aggregation than the engine ignition impact, as shown in Figure 3. Therefore, the gear meshing impact is mostly contained in the component with a high *Q*-factor. To accurately separate the gear vibration signals, an optimized resonance-based signal sparse decomposition (ORSSD) algorithm based on the meshing frequency energy ratio (MF–ER) is proposed and introduced in detail for gear signal identification.

#### 2.2.1. Resonance-Based Signal Sparse Decomposition

The quality factor *Q*, defined as the center frequency divided by bandwidth, can be used to express the oscillatory behavior of a signal,

(2) $$Q\;=\;\frac{f_{c}}{BW}$$

where BW is the bandwidth and $f_{c}$ is the center frequency. A pulse signal with strong resonance property usually has a higher *Q*-factor.

**Figure 4:**
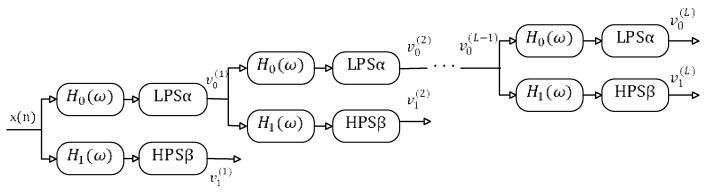
The L layer filter banks. (LPS: low-pass scale, HPS: high-pass scale).

To obtain the corresponding transform coefficients of the RSSD method, TQWT can be used to separate transforms with high and low *Q*-factors. The TQWT is accomplished using two-channel bandpass filter banks, as displayed in Figure 4, where HPS and LPS denote the high-pass scale α and low-pass scale β, which satisfy 0<α<1, 0<β<1, α+β>1. The relationship between α and β is,

(3) β = 2/(Q+1), α = 1−β/r

where r is the redundancy. Therefore, the center frequency $f_{c}$ can be calculated.

(4) $$f_{c}\;=\;\alpha^{j}\frac{2-\beta }{4\alpha }f_{s}\;j\;=\;1,\;\ldots ,\;L$$

According to Equation (4), the center frequency decreases with the increase of the layer L, where $f_{s}$ is the sampling frequency, and the bandwidth BW,

(5) $$BW\;=\;\frac{1}{2}\beta \alpha^{j-1}\pi \;j\;=\;1,\;\ldots ,\;L$$

becomes narrower. In Figure 4, the high-pass and low-pass filters $H_{1}\left(\omega \right)$ and $H_{0}\left(\omega \right)$ can be constructed as follows,

(6) $$H_{1}\left(\omega \right)\;=\;\{\begin{array}{c} 0\;\left|\omega \right|\leq \left(1-\beta \right)\pi \\ \theta \left(\frac{\alpha \pi -\omega }{\alpha +\beta -1}\right)\;\left(1-\beta \right)\pi \leq \left|\omega \right|<\alpha \pi \\ 1\;\alpha \pi \leq \left|\omega \right|<\pi \end{array}$$

(7) $$H_{0}\left(\omega \right)\;=\;\{\begin{array}{c} 1\;\left|\omega \right|\leq \left(1-\beta \right)\pi \\ \theta \left(\frac{\omega +\left(\beta -1\right)\pi }{\alpha +\beta -1}\right)\;\left(1-\beta \right)\pi \leq \left|\omega \right|<\alpha \pi \\ 0\;\alpha \pi \leq \left|\omega \right|<\pi \end{array}$$

where θ(∙) can be expressed by the following function:

(8) $$\theta \left(\omega \right)\;=\;0.5\left(1+cos\omega \right)\sqrt{2-cos\omega },\;\left|\omega \right|\leq \pi$$

It is generally accepted that the vibration signal x(t) can be decomposed into two components with different *Q*-factors [22], which is expressed as,

(9) $$x\left(t\right)=\;x_{1}\left(t\right)+x_{2}\left(t\right)$$

where $x_{1}\left(t\right)$ and $x_{2}\left(t\right)$ denote the two components with high and low Q-factors respectively. To obtain the best expressions of $x_{1}\left(t\right)$ and $x_{2}\left(t\right)$, the morphological component analysis (MCA) [49] was applied to Equation (9). Therefore, the problem can be translated into minimizing the cost function,

(10) $$J\left(w_{1},\;w_{2}\right)\;=\;{\left|\left|x-S_{1}W_{1}-S_{2}W_{2}\right|\right|}_{2}^{2}+\lambda_{1}{\left|\left|W_{1}\right|\right|}_{1}+\lambda_{2}{\left|\left|W_{2}\right|\right|}_{1}$$

where $S_{1}$, $S_{2}$ denote the overcomplete dictionaries for $x_{1}\left(t\right)$, $x_{2}\left(t\right)$; $W_{1}$, $W_{2}$ indicate the wavelet coefficients of $x_{1}\left(t\right)$, $x_{2}\left(t\right)$; and $\lambda_{1}$, $\lambda_{2}$ are the regularization parameters. To solve Equation (10), the split augmented Lagrangian shrinkage algorithm [50] was used to iterate and update the wavelet coefficients $W_{1}$ and $W_{2}$.

If the cost function achieves the minimum when the corresponding coefficients are $W_{1}^{*}$ and $W_{2}^{*}$, then the components with different *Q*-factors can be obtained.

(11) $${\hat{x}}_{1}\;=\;S_{1}W_{1}^{*},\;{\hat{x}}_{2}\;=\;S_{2}W_{2}^{*}$$

#### 2.2.2. Parameter Selection Problem

According to the analysis above, six parameters need to be selected for the RSSD algorithm, that is the quality factors, decomposition layers, and redundancies of both high- and low-resonance parts ($Q_{1},\;Q_{2},\;L_{1},\;L_{2},\;r_{1},\;r_{2}$). Among these parameters, the selection of the quality factors plays the most significant role in the decomposition accuracy, as the quality factors reflect the oscillatory behaviors of the decomposed components [44]. Suppose $Q_{1}$, $Q_{2}$ have been determined, the redundancies $r_{1}$, $r_{2}$ will affect the sparsity of adjacent frequency responses. Therefore, the values of the redundancies cannot be too big or too small. Based on the research in [15], the redundancies $r_{1}$ and $r_{2}$ are all chosen as 3 in our algorithm. As for the decomposition layers $L_{1}$ and $L_{2}$, the maximum values are employed to guarantee all signal information is contained in the sub-bands. The maximum decomposition layers can be calculated by the following equation [21],

(12) $$L_{max}\;=\;\left[\frac{log\left(\frac{N}{4\left(Q+1\right)}\right)}{log\left(\frac{Q+1}{Q+1-2/r}\right)}\right]$$

where *N* denotes the data length and [∙] represents the rounding operation.

To sum up, the biggest obstacle in implementing the RSSD algorithm is the selection of the appropriate quality factors, which is of great significance to the accuracy of RSSD. To solve this problem, a parameter optimization strategy based on the meshing frequency energy ratio (MF–ER) is proposed to adaptively determine the quality factors of both high- and low-resonance components.

#### 2.2.3. Parameter Optimization Based on Meshing Frequency Energy Ratio

To adaptively select the quality factors $Q_{1}$ and $Q_{2}$, the ranges of them are firstly determined as $Q_{1}\in \left[4,\;12\right]$ and $Q_{2}\in \left[1,\;3\right]$ [51]. Then the RSSD algorithm with different Q combinations is applied to the original gear vibration data x(t). Since the gear vibration is mostly contained in the component with a high *Q*-factor, the high resonance component is used and denoted as $x_{1}\left(t\right)$, the analytical signal of $x_{1}\left(t\right)$ is,

(13) $$z_{1}\left(t\right)\;=\;x_{1}\left(t\right)+i\bar{H_{1}}\left(t\right)$$

where $\bar{H_{1}}\left(t\right)$ indicates the Hilbert transform of $x_{1}\left(t\right)$. Thence, the envelope waveform is calculated by taking the absolute value of $z_{1}\left(t\right).$

(14) $$e\left(t\right)\;=\;\left|z_{1}\left(t\right)\right|\;=\;\sqrt{{\left(x_{1}\left(t\right)\right)}^{2}+{\left(\bar{H_{1}}\left(t\right)\right)}^{2}}$$

By applying Fourier transform to e(t), the envelope spectrum E(f) of the high resonance component is obtained. Based on it, the meshing frequency energy ratio (MF–ER) can be defined, which demonstrates the proportion of vibration energy contributed by gear meshing frequency. The gear meshing frequency is denoted as $f_{m}$. Therefore, MF–ER can be calculated using the following equation,

(15) $$\mathrm{MF}–\mathrm{ER}\;=\;\frac{\sum_{1}^{K}{\left[E\left(f_{Km}\right)\right]}^{2}}{\sum_{0}^{f_{s}/2}{\left[E\left(f\right)\right]}^{2}}$$

where $f_{s}$ represents the sampling rate and K represents the number of meshing frequency harmonics. According to [46,52,53], only the first three harmonics are concerned. The reason is that the first three meshing frequency harmonics contain most of the energy. Therefore, K is set to be 3 in our study. It can be seen that MF–ER is sensitive to the gear meshing frequency. It can be used as a novel index to measure periodic impulses. A bigger MF–ER value implies the better performance of the RSSD algorithm in gear signal extraction.

Using the MF–ER index, the optimal Q-factors are selected by iterating the $Q_{1}$ and $Q_{2}$ values in the ranges mentioned above. In our method, the optimization step size is chosen as 0.5 according to [51,54].

### 2.3. The Proposed AR-ORSSD Algorithm

Motivated by the AR model and ORSSD method based on the MF–ER index, the AR-ORSSD algorithm is presented in our research for gear vibration signal identification. The main steps of the AR-ORSSD algorithm are summarized as follows:

(1) Remove the normal gear meshing vibration using the AR model;
(2) Determine the ranges of $Q_{1}$, $Q_{2}$, $Q_{1}\in \left[4,\;12\right]$, $Q_{2}\in \left[1,\;3\right]$, in steps of 0.5;
(3) Perform the RSSD operation;
(4) Calculate the MF–ER value for each combination of $\left[Q_{1},\;Q_{2}\right]$;
(5) Obtain the optimal Q-factors when MF–ER achieves the maximum;
(6) Implement the RSSD with the optimal Q-factors;
(7) Identify the gear vibration signal with the optimized RSSD method.

**Figure 5:**
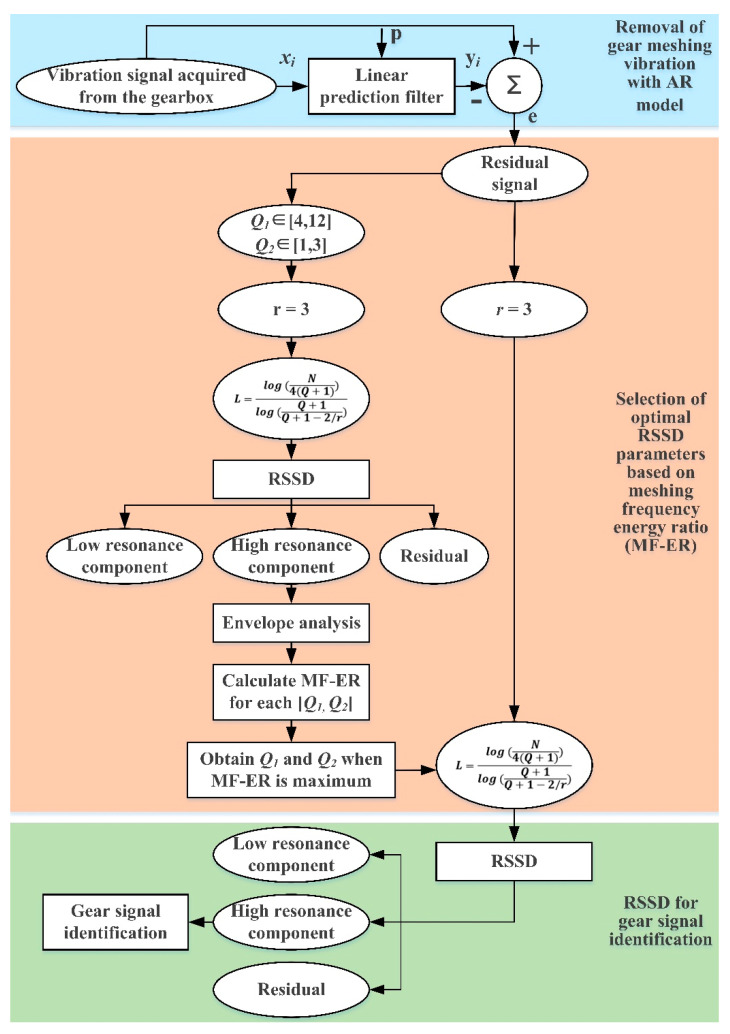
The framework of the proposed auto-regression model-based optimized resonance-based signal sparse decomposition (AR-ORSSD) algorithm.

Figure 5 illustrates the framework of the proposed AR-ORSSD algorithm.

## 3. Simulated Signal Analysis

In this section, both the gear vibration model and the engine ignition impact model are established to validate the effectiveness of the proposed algorithm. The result obtained by each step of the method is displayed. Comparisons are made between the results with and without the AR model operation, which can prove the necessity of adopting the AR model. Besides, the proposed method is also compared with the popular RSSD algorithm optimized by genetic algorithm (GA).

In general, the vibration signal acquired from a perfect gear transmission is modeled by amplitude and phase modulations accompanied with meshing impacts, which can be described by the following equation [55],

(16) $$\begin{array}{ll} x_{gear}\left(t\right)\;= & \left[1+A_{g}cos\left(2\pi f_{r,p}t\right)\right]cos\left[2\pi f_{m}t+B_{g}cos\left(2\pi f_{r,p}t\right)\right]\\ & +\left[1+A_{g}cos\left(2\pi f_{r,g}t\right)\right]cos\left[2\pi f_{m}t+B_{g}cos\left(2\pi f_{r,g}t\right)\right]\\ & +\sum_{k=0}^{K}\;A_{m}e^{-\beta_{m}\left(t-t_{k,\;m}\right)}cos\left[2\pi f_{mr}\left(t-t_{k,\;m}\right)\right]u\left(t-t_{k,\;m}\right) \end{array}$$

where $f_{m}$ is the gear meshing frequency, $f_{r,p}$ and $f_{r,g}$ are the rotating frequencies of the pinion and gear, and $f_{mr}$ is the resonance frequency excited by gear meshing impact. $A_{g}$ and $B_{g}$ are the magnitudes of the amplitude and phase modulations, respectively. $A_{m}$ denotes the amplitude of the impulses due to meshing impacts. $\beta_{m}$ represents the damping characteristic frequency and $t_{k,\;m}$ is the time of occurrence of the kth impulse.

Considering the characteristics of the ignition impact vibration of the engine, it can be recognized as periodic impulses, which can be modeled as,

(17) $$x_{engine}\left(t\right)\;=\;\overset{N}{\underset{n\;=\;0}{\sum }}A_{e}e^{-\beta_{e}\left(t-t_{n,\;e}\right)}cos\left[2\pi f_{er}\left(t-t_{n,\;e}\right)\right]u\left(t-t_{n,\;e}\right)$$

where $A_{e}$ is the amplitude of the ignition impulses of the engine, $\beta_{e}$ is the structural damping characteristic frequency of the ignition impact, $f_{er}$ is the resonance frequency induced by the engine ignition impact, and $t_{n,\;e}$ denotes the time of occurrence of the mth impulse.

In our simulation, the pinion and gear have 13 and 35 teeth respectively. The sampling frequency is set as 20,000 Hz. The ignition frequency $f_{en}$ is set as 20 Hz. The other parameters are listed in Table 1.

**Figure 6:**
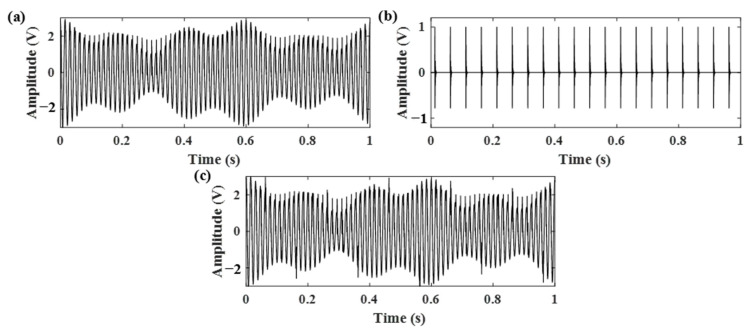
Simulated signals: (**a**) gear vibration signal, (**b**) engine ignition signal, (**c**) compound signal of the gear and engine.

**Figure 7:**
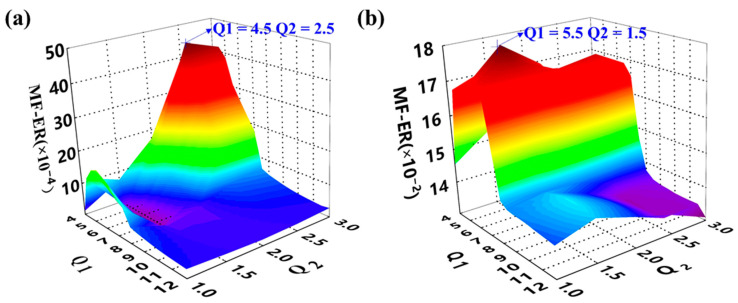
The meshing frequency energy ratio (MF–ER) values with different Q-factors of the simulated signal. (**a**) The result without pre-whitening using AR model; (**b**) The result of the proposed AR-ORSSD algorithm. (The arrows in the picture indicate the Q-factors with the maximum MF–ER).

The simulated gear vibration signal, the engine ignition signal, and the compound signal are shown in Figure 6. It can be seen that in the compound signal, the gear meshing impact is submerged by gear meshing vibration and engine ignition signal. To separate the gear meshing impact, the optimized RSSD method was employed. According to our analysis, the MF–ER values changing with different *Q*-factors with and without the AR model operation were calculated. The corresponding results are plotted in Figure 7. Therefore, the optimal *Q*-factors are (4.5, 2.5) and (5.5, 1.5), respectively.

**Figure 8:**
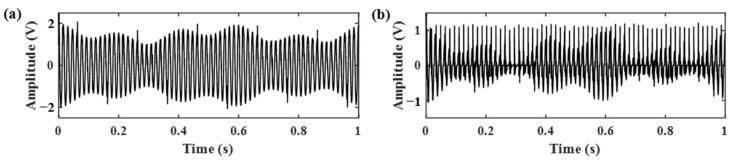
The decomposition results of RSSD using parameters obtained from Figure 7a. (**a**) The high-resonance component; (**b**) the low-resonance component.

Firstly, the RSSD method was performed using parameters obtained without the AR model pre-whitening process. The data length is chosen as 20,000. The obtained high- and low-resonance components are displayed in Figure 8. According to the results, the high resonance component contains most of the gear meshing vibration, the gear meshing impacts are still hard to identify.

**Figure 9:**
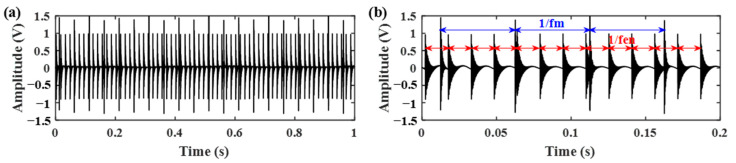
The AR residual signal. (**a**) The time waveform; (**b**) The enlarged time waveform.

**Figure 10:**
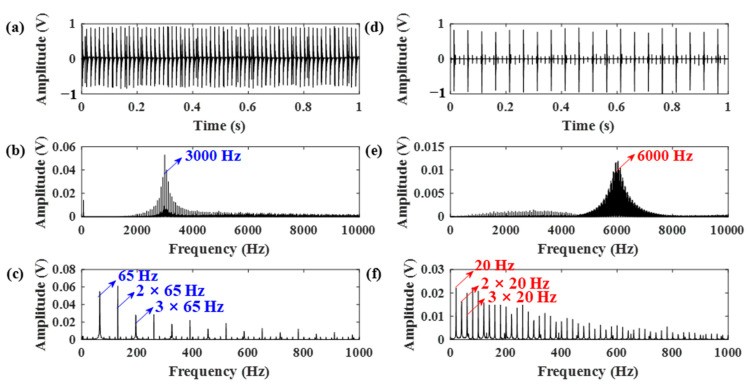
The decomposition results of the RSSD using the proposed AR-ORSSD algorithm. (**a**–**c**) The high resonance component, its frequency spectrum, and envelope spectrum; (**d**–**f**) the low resonance component, its frequency spectrum, and envelope spectrum (the arrows in blue indicate the characteristic frequencies of the gear while the arrows in red indicate the characteristic frequencies of the engine).

Using the proposed AR-ORSSD algorithm, the AR model was first used to remove the normal gear meshing vibration, and the obtained residual signals are shown in Figure 9. It can be seen from the enlarged time domain signal that the gear meshing vibration is almost eliminated, with only gear meshing impact and engine ignition signal remaining. Then, the residual signal was subjected to RSSD with the optimal *Q*-factors (5.5, 1.5). The decomposition results are demonstrated in Figure 10. In Figure 10, the original signal is successfully separated into gear meshing impact and engine ignition signal and they are contained in high- and low-resonance components, respectively.

**Figure 11:**
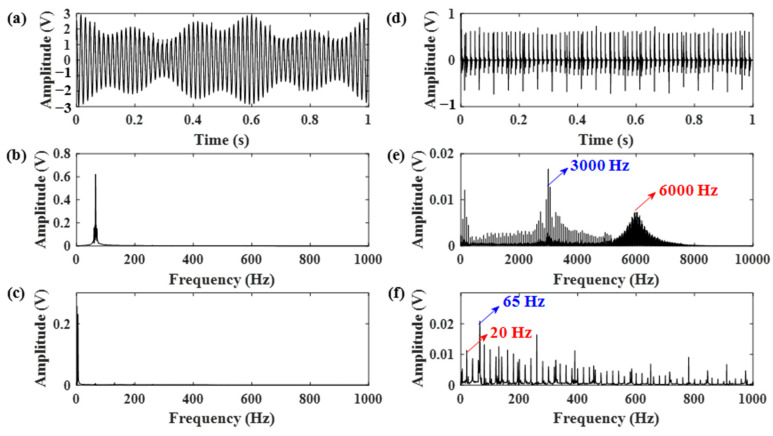
The decomposition results of the RSSD optimized by genetic algorithm. (**a**–**c**) The high-resonance component, its frequency spectrum, and envelope spectrum; (**d**–**f**) the low-resonance component, its frequency spectrum, and envelope spectrum (the arrows in blue indicate the characteristic frequencies of the gear while the arrows in red indicate the characteristic frequencies of the engine).

To further verify the performance of the proposed algorithm, the popular ORSSD method optimized by the GA algorithm was used in our experiment as a comparison. The GA-based RSSD algorithm has achieved perfect performance in some researches [31,34]. In our simulation, the optimal parameters optimized by the GA algorithm are obtained and shown in Table 2. The corresponding decomposition results are illustrated in Figure 11. The results show that the high-resonance component mainly includes the gear meshing vibration; the gear meshing impact and the engine ignition signal are all contained in the low-resonance component. Therefore, the gear meshing impact cannot be easily identified.

## 4. Experimental Verification

In this section, field experiments were performed with a forklift to identify the gear vibration signal in the engine-gearbox system to validate the performance of the proposed AR-ORSSD algorithm. The forklift was chosen as the experimental subject because of three reasons: (1) the forklift usually works under heavy load conditions. Researches have shown that the intensity of the gear meshing impact is positively correlated with the load applied to the gear [56,57]. (2) Vibration and noise have long been an intractable issue in the forklift industry. Researches have investigated that there are risks of whole-body vibration for lower back pain among forklift truck drivers [58]. (3) Too much vibration causes damage to the components, lessening the service life of forklifts.

**Figure 12:**
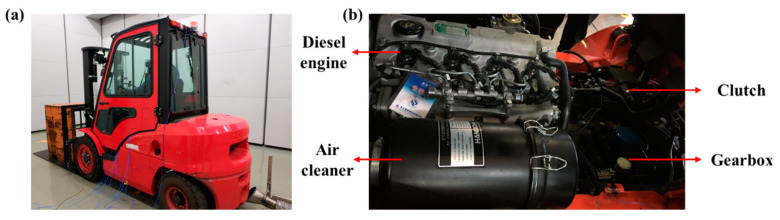
The schematic diagrams of (**a**) the forklift for the experiment, (**b**) the transmission system.

The model diagram of the forklift used in our experiment is illustrated in Figure 12a, Figure 12b demonstrates the schematic diagram of the transmission system. In our experiment, two accelerometers are installed on the engine and gearbox, respectively. The vibration signals of the engine and gearbox are acquired for further investigation. The sampling frequency used in our experiments is 12,800 Hz.

In our experiment, the engine rotating speed was set at 703.2 rpm. The tooth number of the pinion and gear were 17 and 29, respectively. Therefore, the meshing frequency can be calculated as 199.24 Hz. The cylinder number N=4, and the engine stroke constant D=2. The relationship between the engine rotating speed v and ignition frequency $f_{en}$ is expressed by the following equation,

(18) $$f_{en}\;=\;\frac{Nv}{60D}$$

Therefore, the ignition frequency $f_{en}$ can be calculated as 23.44 Hz.

**Figure 13:**
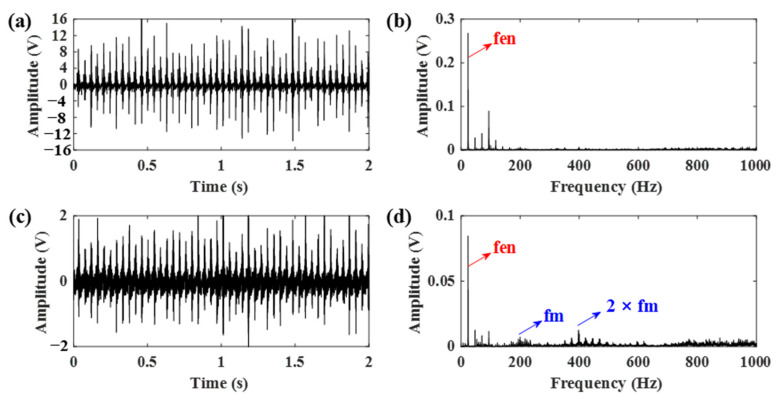
Vibration signals acquired from the engine and gearbox. (**a**,**b**) The time domain signal and corresponding frequency spectrum of the engine; (**c**,**d**) the time domain signal and corresponding frequency spectrum of the gearbox (the arrows in blue indicate the characteristic frequencies of the gear while the arrows in red indicate the characteristic frequencies of the engine).

During the experiment, the forklift was driven by a professional driver with a 2.5 ton load. The obtained vibration signals of both engine and gearbox are displayed in Figure 13. By comparing the two frequency spectrums, it can be seen that the ignition impact of the engine is dominant in the frequency domain, making the gear meshing frequency hard to be identified. Moreover, little information about gear meshing can be easily extracted from the time-domain signal of the gear vibration.

**Figure 14:**
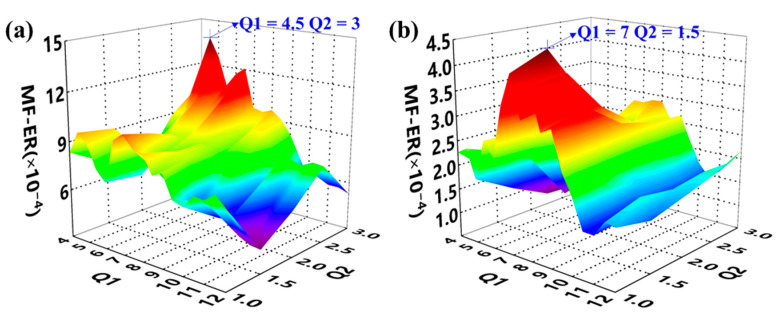
The MF–ER values with different Q-factors of the gear vibration signal. (**a**) The result without pre-whitening using AR model; (**b**) the result of the proposed AR-ORSSD algorithm (the arrows in the picture indicate the Q-factors with the maximum MF–ER).

Motivated by the proposed method, the AR model was applied to the gear vibration signal to remove the normal gear meshing vibration. Based on the AR residual signal, the optimal Q-factors were selected by calculating the MF–ER values of each combination of $\left(Q_{1},\;Q_{2}\right)$. The outcome of the MF–ER values changing with different Q-factors is expressed by 3D graphs shown in Figure 14. Both results obtained from the proposed algorithm and the ORSSD method without the AR model are provided. It can be seen that the optimal Q-factors of the two situations are (4.5, 3) and (7, 1.5).

**Figure 15:**
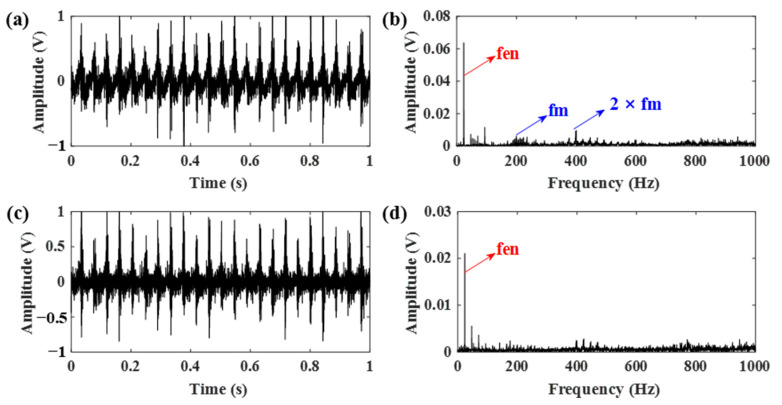
The decomposition results of the RSSD using parameters obtained from Figure 14a. (**a**,**b**) The high-resonance component and its frequency spectrum; (**c**,**d**) the low-resonance component and its frequency spectrum (the arrows in blue indicate the characteristic frequencies of the gear while the arrows in red indicate the characteristic frequencies of the engine).

**Figure 16:**
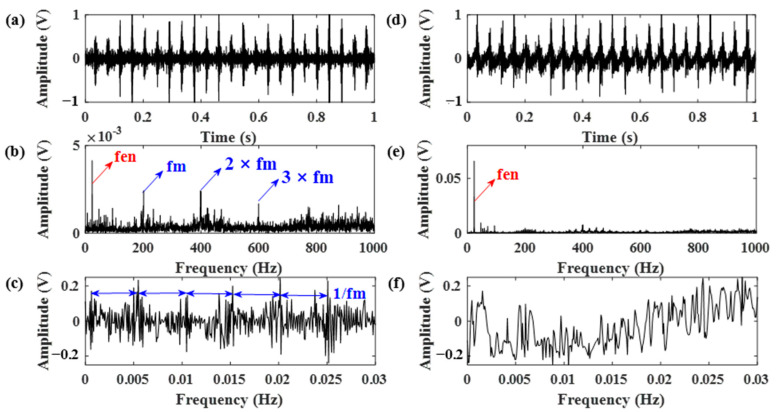
The decomposition results of the RSSD using the proposed AR-ORSSD algorithm. (**a**–**c**) The high-resonance component, its frequency spectrum and enlarged high-resonance component; (**d**–**f**) the low-resonance component, its frequency spectrum, and enlarged high-resonance component (the arrows in blue indicate the characteristic frequencies of the gear while the arrows in red indicate the characteristic frequencies of the engine).

Therefore, the gear vibration data was subjected to the RSSD method using the obtained optimal Q-factors. The data length was chosen as 12,800. The decomposition results using parameters obtained from both Figure 14a,b are shown in Figure 15 and Figure 16. According to Figure 15, the RSSD method cannot separate the gear meshing frequency from the strong ignition impact signal of the engine without pre-whitening using the AR model. However, it can be seen from Figure 16 that the gear meshing frequency can be successfully identified from the high resonance component using the proposed AR-ORSSD algorithm. The meshing frequency and its harmonics can be clearly found in the frequency spectrum of the high resonance component. In addition, the meshing impulses can be extracted from the enlarged high-resonance component.

**Figure 17:**
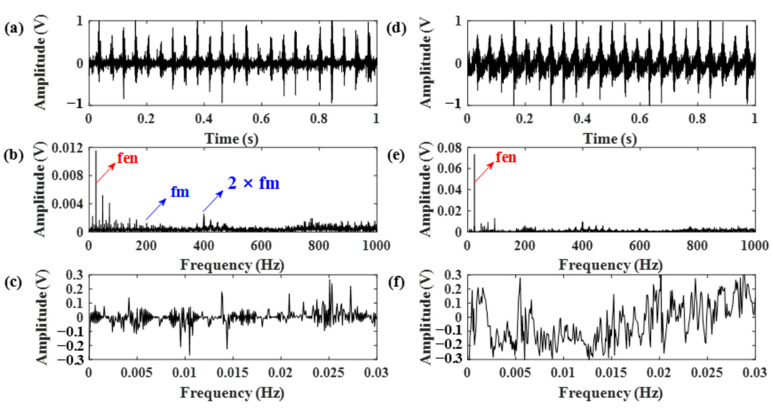
The decomposition results of the RSSD optimized by GA. (**a**–**c**) The high-resonance component, its frequency spectrum, and enlarged high-resonance component; (**d**–**f**) the low-esonance component, its frequency spectrum, and enlarged high-resonance component (the arrows in blue indicate the characteristic frequencies of the gear while the arrows in red indicate the characteristic frequencies of the engine).

To better prove the effectiveness of the proposed algorithm, the same data was also subjected to the RSSD method optimized by the GA algorithm. The obtained parameters are listed in Table 3. Using these parameters, the RSSD decomposition results were demonstrated in Figure 17. Compared with the results in Figure 16, the decomposition results of the RSSD optimized by GA cannot successfully identify the gear meshing impact as the proposed AR-ORSSD algorithm does.

## 5. Conclusions

In engine-gearbox systems, the ignition impact of the engine is significant. To identify the gear meshing frequency under strong interferences, a resonance-based signal sparse decomposition method (AR-ORSSD) that uses auto-regression (AR) as a pre-whitening step to eliminate the normal gear meshing vibration is introduced here for the first time. The main contributions of this paper are summarized as follows.

(1) The main idea of this paper is that the gear meshing impact has better frequency aggregation than the engine ignition impact. Therefore, the RSSD algorithm is introduced.
(2) The biggest innovation of this paper is that we define the MF–ER index and introduce it into the RSSD algorithm to adaptively choose the optimal *Q*-factors, which can improve the accuracy of the separation results.
(3) Due to the interferences of the normal gear meshing vibration, the use of the RSSD algorithm alone cannot achieve perfect results. Therefore, the AR model is used as a pre-processing step to eliminate the normal gear meshing vibration.
(4) Both simulated signals and experimental signals acquired from the engine-gearbox system in a forklift validate the effectiveness of the proposed algorithm.
(5) Both simulated signals and experimental signals validate the necessity of adopting the AR model.
(6) Through comparison with the GA-based RSSD method, it is indicated that the AR-ORSSD algorithm achieves superior performance in identifying gear vibration signals especially when under strong interferences.

This paper mainly focuses on identifying the gear meshing impact under the interference of the engine ignition impact. The proposed algorithm can be extended to solve other problems. For example, for the compound fault diagnosis of the gear and bearing in a gearbox, the vibration impacts excited by gear and bearing defects also have different oscillatory behaviors: the proposed method can be used to solve the problem.

In this preliminary study, the proposed method was tested using the engine-gearbox system of a forklift under constant speed and load conditions. Further research with the engine-gearbox systems of other industrial equipment such as cars and trains can be considered in the future. Besides, tests under variable speeds and load conditions can also be analyzed in our future work.

## Figures and Tables

**Table 1:** The required parameters for the simulation.

Parameters	Value	Parameters	Value
$A_{g}$	0.5	$\beta_{m}$	1000
$B_{g}$	0.2	$\beta_{e}$	600
$A_{m}$	1	$f_{en}$	20
$f_{r,p}$	5	$f_{mr}$	3000
$f_{r,g}$	2	$f_{er}$	6000
$f_{m}$	65	$t_{k,\;m}$	0.015
$A_{e}$	1	$t_{n,\;e}$	0.05

**Table 2:** The RSSD parameters optimized by the GA algorithm.

$Q_{1}$	$Q_{2}$	$r_{1}$	$r_{2}$
4.65	1	5.05	6.44

**Table 3:** The RSSD parameters optimized by the GA algorithm.

$Q_{1}$	$Q_{2}$	$r_{1}$	$r_{2}$
9.21	1.74	9.83	3.88

## Data Availability

Not applicable.

## Nomenclature

$x_{t},\;x_{t-i}$	Data points at time t, t−i respectively, V
n	Model order
p	The optimal model order
$a_{i}$	ith coefficient of the AR model
$\varepsilon_{t}$	Residual error at time t, V
Q	Quality factor
$f_{c}$	Center frequency, Hz
BW	Bandwidth, Hz
α	High-pass scale
β	Low-pass scale
r	Redundancy
L	Decomposition layer
$f_{s}$	Sampling frequency, Hz
$H_{1}\left(\omega \right),\;H_{0}\left(\omega \right)$	High-pass and low-pass filters
ω	Angle, rad
θ(∙)	Function, $\theta \left(\omega \right)\;=\;0.5\left(1+cos\omega \right)\sqrt{2-cos\omega },\;\left|\omega \right|\leq \pi$
x(t)	Vibration signal, V
$x_{1}\left(t\right),\;x_{2}\left(t\right)$	High and low resonance components, V
$S_{1},\;S_{2}$	The overcomplete dictionaries for $x_{1}\left(t\right),\;x_{2}\left(t\right)$
$W_{1},\;W_{2}$	The wavelet coefficients of $x_{1}\left(t\right)$, $x_{2}\left(t\right)$
$\lambda_{1},\;\lambda_{2}$	The regularization parameters of $x_{1}\left(t\right)$, $x_{2}\left(t\right)$
$W_{1}^{*},\;W_{2}^{*}$	The wavelet coefficients of $x_{1}\left(t\right)$ and $x_{2}\left(t\right)$ when cost function achieves the minimum
${\hat{x}}_{1},\;{\hat{x}}_{2}$	The optimal high and low resonance components, V
$Q_{1},\;Q_{2}$	Quality factors of $x_{1}\left(t\right)$ and $x_{2}\left(t\right)$
$L_{1},\;L_{2}$	Decomposition layers of $x_{1}\left(t\right)$ and $x_{2}\left(t\right)$
$r_{1},\;r_{2}$	Redundancies of $x_{1}\left(t\right)$ and $x_{2}\left(t\right)$
N	The data length
[∙]	The rounding operation
*MF–ER*	Meshing frequency energy ratio
$\bar{H_{1}}\left(t\right)$	The Hilbert transform of $x_{1}\left(t\right)$, V
$z_{1}\left(t\right)$	The analytical signal of $x_{1}\left(t\right)$, V
e(t)	The envelop waveform of $z_{1}\left(t\right)$, V
E(f)	The envelop spectrum of $x_{1}\left(t\right)$, V
$f_{m}$	Gear meshing frequency, Hz
K	The number of meshing frequency harmonics
$f_{r,p},\;f_{r,g}$	The rotating frequencies of the pinion and gear, Hz
$f_{mr}$	The resonance frequency excited by gear meshing impact, Hz
$A_{g},\;B_{g}$	The magnitudes of the amplitude and phase modulations, V
$A_{m}$	The amplitude of the impulses due to meshing impacts, V
$\beta_{m}$	Damping characteristic frequency, Hz
$f_{en}$	Ignition frequency, Hz
$t_{k,\;m}$	The time of occurrence of the *k*th impulse, s
$A_{e}$	The amplitude of the ignition impulses of the engine, V
$\beta_{e}$	The structural damping characteristic frequency of the ignition impact, Hz
$f_{er}$	The resonance frequency induced by the engine ignition impact, Hz
$t_{n,\;e}$	The time of occurrence of the *m*th impulse, s
$N^{\prime }$	The cylinder number
D	The engine stroke constant
v	Engine rotating speed, rpm
